# MRI pattern characterization of cerebral cardioembolic lesions following atrial fibrillation ablation

**DOI:** 10.3389/fcvm.2024.1327567

**Published:** 2024-01-24

**Authors:** Andrea Saglietto, Eleonora Bertello, Marina Barra, Ilenia Ferraro, Chiara Rovera, Fulvio Orzan, Gaetano Maria De Ferrari, Matteo Anselmino

**Affiliations:** ^1^ Division of Cardiology, Cardiovascular and Thoracic Department, “Città della Salute e della Scienza” Hospital, Turin, Italy; ^2^ Department of Medical Sciences, University of Turin, Turin, Italy; ^3^ Division of Cardiology, Santa Croce e Carle Hospital, Cuneo, Italy; ^4^ Department of Cardiology, Civic Hospital of Chivasso, Chivasso, Italy

**Keywords:** stroke, imaging, cardioembolic, MRI pattern, atrial fibrillation, ablation

## Abstract

**Background:**

Recognizing etiology is essential for treatment and secondary prevention of cerebral ischemic events. A magnetic resonance imaging (MRI) pattern suggestive of an embolic etiology has been described but, to date, there are no uniformly accepted criteria.

**Aim:**

The purpose of the study is to describe MRI features of ischemic cerebral lesions occurring after transcatheter ablation of atrial fibrillation (AF).

**Methods:**

A systematic review and meta-analysis of studies performing brain imaging investigations before and after AF transcatheter ablation was performed. The incidence of cerebral ischemic lesions after AF transcatheter ablation was the primary endpoint. The co-primary endpoints were the prevalence of the different neuroimaging features regarding the embolic cerebral ischemic lesions.

**Results:**

A total of 25 studies, encompassing 3,304 patients, were included in the final analysis. The incidence of ischemic cerebral lesions following AF transcatheter ablation was 17.2% [95% confidence interval (CI) 12.2%–23.8%], of which a minimal fraction was symptomatic [0.60% (95% CI 0.09%–3.9%)]. Only 1.6% of the lesions (95% CI 0.9%–3.0%) had a diameter >10 mm, and in 20.5% of the cases the lesions were multiple (95% CI 17.1%–24.4%). Brain lesions were equally distributed across the two hemispheres and the different lobes; cortical location was more frequent [64.0% (95% CI 42.9%–80.8%)] while the middle cerebral artery territory was the most involved 37.0% (95% CI 27.3–48.0).

**Conclusions:**

The prevailing MRI pattern comprises a predominance of small (<10 mm) cortical lesions, more prevalent in the territory of the middle cerebral artery.

## Introduction

Stroke is a leading cause of mortality and long-term disability (1); recognizing the underlying cause of stroke is relevant for treatment, prognosis, and secondary prevention (2). However, about 25% of strokes are defined “cryptogenic” because the etiology remains unknown in spite of exhaustive investigations (3). Hart et al. recently proposed to call these types of lesions “embolic strokes of undetermined source” (ESUS) (4), considering that the vast majority recognizes an embolic etiology. Detecting a potential mechanism would, indeed, be relevant, when therapeutic options aiming at preventing recurrences are available, such as the initiation of oral anticoagulation in case of atrial fibrillation (AF) or percutaneous closure in case of suspected paradoxical embolism through a patent foramen oval (PFO).

With the development of computerized tomography (CT) and magnetic resonance imaging (MRI) techniques, a specific imaging pattern of brain lesions for every etiologic type of acute stroke has been hypothesized. A number of studies have concluded that in cardioembolic strokes, lesions are mostly multiple and cortical in location (5–7), but a systematic description of the neuroimaging features associated with a cardioembolic genesis of ischemic strokes is presently lacking. In the past two decades, silent cerebral lesions have been described at MRI following AF transcatheter ablation (8). These lesions have been carefully identified by comparing imaging before and after the procedure with MRI diffusion-weighted imaging (DWI) sequences, the most sensitive technique for the detection of acute cerebral ischemia (9).

Because during an uncomplicated transcatheter AF ablation the patient does not experience hemodynamic compromise, we hypothesize that the pathophysiological mechanism underlying brain lesions is embolic, and not related to hypoperfusive genesis. The major mechanisms involved include endothelium damage at transseptal puncture, conventional clotting (e.g., from the groin) and crossed emboli in the iatrogenic interatrial septum defect, and thermal thrombus formation in the left atrium (charring and gas embolism). In addition, during a transcatheter ablation the introduction of bulky devices, such as multielectrode catheters, and balloons carries a risk of air embolism (10).

In the present study we conducted a systematic review and meta-analysis to describe the neuroimaging features of newly formed cardioembolic lesions after AF transcatheter ablation.

## Methods

This work was conducted following the Preferred Reporting Items for Systematic reviews and Meta-Analyses (PRISMA) guidelines (11).

### Search strategy

Pertinent articles were searched in MEDLINE/PubMed with MeSH strategy, using the following terms: ((cerebral lesion* OR stroke OR silent cerebral lesion OR SCI embolism) AND cerebral MRI AND (Atrial fibrillation ablation OR Fib ablation OR AF ablation)). The search was ended on 30 January 2023.

### Study selection and data extraction

Two independent reviewers (EB, MB) screened the retrieved citations through title and/or abstract. When potentially pertinent, the studies were appraised as complete reports according to the following inclusion/exclusion criteria:

• They reported the absolute number of new ischemic cerebral lesions that occurred after AF transcatheter ablation; this implies that the included studies were designed to perform cerebral MRI scan prior to and immediately after the ablation procedure.
• They reported at least one of the evaluated characteristics of the ischemic cerebral lesions (please refer to the following section regarding the specific study endpoints for detailed description of evaluated features).

Exclusion criteria were non-human setting; duplicate reporting (in which case, the manuscript reporting the largest sample of patients was selected).

Two independent, unblinded reviewers (EB and MB) abstracted the following data on prespecified forms: authors, journal, year of publication, baseline clinical and interventional features, cerebral MRI protocol, and neuroimaging features. Data collection was conducted by mutual agreement and all potential disagreement was resolved by a third reviewer (AS).

### Study endpoints

The incidence of new cerebral ischemic lesions after AF transcatheter ablation was the primary endpoint. The co-primary endpoints were the prevalence of the following neuroimaging features regarding new cerebral ischemic lesions:

• Lesion dimension: size > 10 mm;
• Multiple lesions;
• Left vs. right hemisphere location;
• Cortical, subcortical (white matter and/or basal ganglia), or cerebellar location;
• Involved anatomical lobe: frontal, parietal, occipital, temporal;
• Involved vascular territory: anterior cerebral artery (ACA), middle cerebral artery (MCA), posterior cerebral artery (PCA), and border zone (BZ).

The secondary endpoint was the incidence of symptomatic cerebral ischemic events (stroke/transient ischemic attack).

### Statistical analysis

The baseline characteristics of the pooled study populations were reported as median values and their interquartile ranges (IQRs). The meta-analysis of the proportions (crude incidence of new cerebral ischemic lesions and prevalence of the prespecified neuroimaging features) was performed using a generalized linear mixed models (12) under a random-effect framework and the results were reported together with the corresponding 95% confidence interval (CI). Cochrane *I*^2^ test was used to investigate heterogeneity, with *I*^2^ values of 25%, 50%, and 75% representing, respectively, mild, moderate, and extensive heterogeneity. Statistical analyses were performed with R version 4.0.0 (R Foundation for Statistical Computing, Vienna, Austria) and *p*-values less than 0.05 were considered statistically significant.

## Results

**Figure 1:**
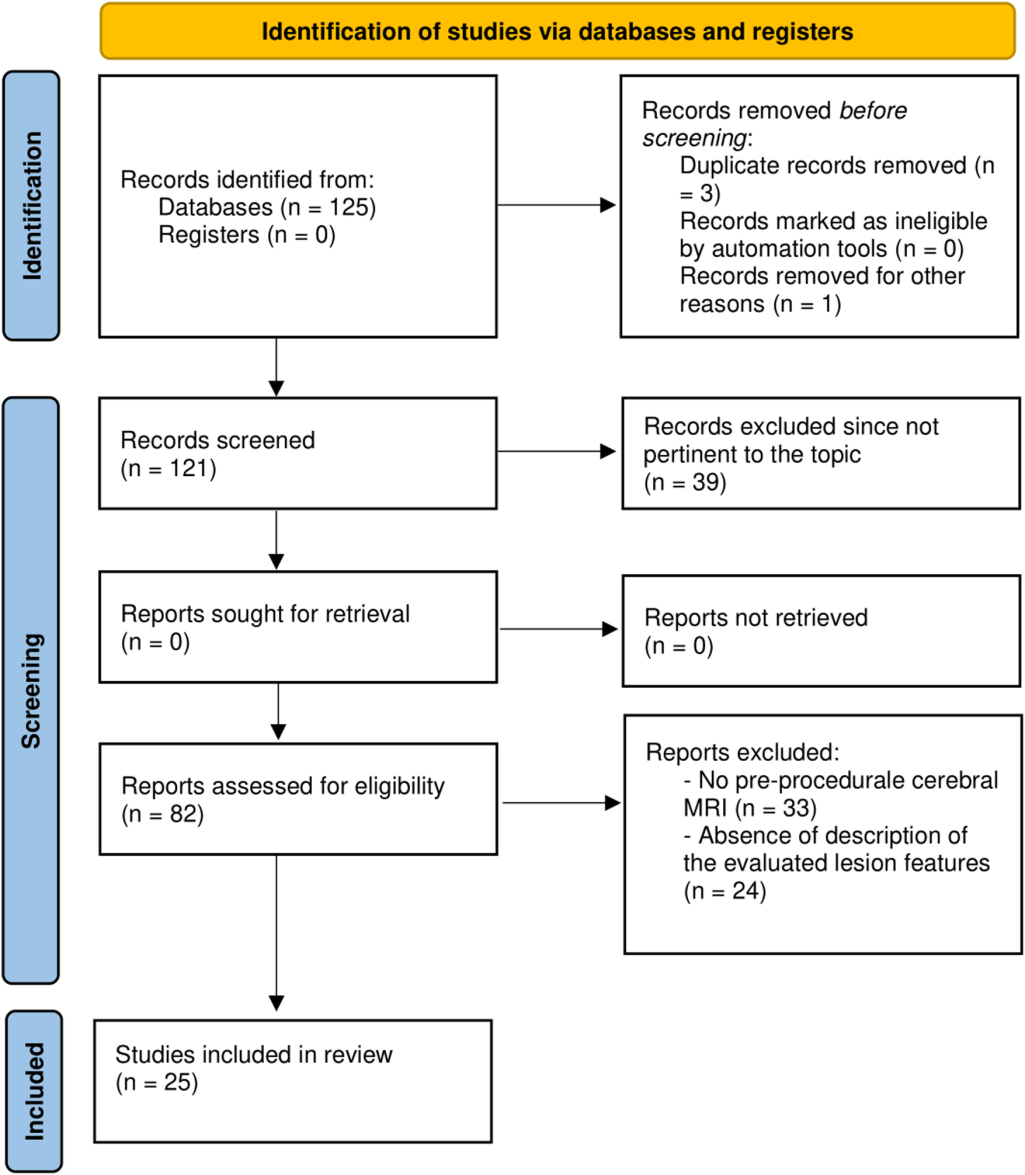
PRISMA flowchart of the study selection process.

The initial search retrieved 125 studies. Among these, three studies were removed because of duplication, and one study was not assessed for eligibility because of the unavailability of the English translation. Given that 39 studies were not pertinent to the topic of the meta-analyses, 82 studies were assessed for eligibility, and 25 eventually included (13–37). Figure 1 reports the detailed PRISMA flowchart of the selection process. The resulting meta-analytic population encompassed 3,304 patients who had undergone AF transcatheter ablation and pre- and post-procedure cerebral MRI scans to detect new ischemic lesions. More details regarding the included studies, in particular concerning the adopted cerebral MRI protocol, the ablation technique, and the energy source are reported in Table 1. The AF type was paroxysmal in the majority of the patients (69%, IQR 59%–97%). The median age was 61 (IQR 58–63) years with a nearly 2:1 male-to-female ratio (males 68%, IQR 63–75).

**Table 1:** General characteristics of the included studies.

Study	Patients	Study-specific MRI definition of cerebral ischemic lesions	Number of lesions	Ablation details
Lickfett et al. (13)	20	DWI/FLAIR/TSE sequences	3	RF, point-by-point
Gaita et al. (14)	232	DWI EPI sequences	34	RF, point-by-point
Neumann et al. (24)	89	DWI EPI sequences	7	RF, point-by-point (49.4%)/Cryobaloon, single shot (50.6%)
Gaita et al. (31)	108	DWI/FLAIR sequences	36	RF, point-by-point (66.7%)/Cryobaloon, single shot (33.3%)
Deneke et al. (32)	86	DWI EPI sequences	119	RF, point-by-point
Siklódy et al. (33)	74	DWI EPI sequences	30	RF, point-by-point (68.9%)/Cryobaloon, single shot (31.1%)
Scaglione et al. (34)	80	DWI EPI sequences	7	RF, point by point
Rillig et al. (35)	70	FLAIR/DWI EPI sequences/T1	16	RF, point-by-point
Sramko et al. (36)	58	DWI sequences	1	RF, point-by-point
Herm et al. (37)	37	DWI sequences	56	RF, point-by-point
Verma et al. (15)	60	DWI sequences	1	RF, point-by-point
Martinek (16)	131	DWI EPI sequences	25	RF, point-by-point
Haeusler et al. (17)	37	DWI EPI sequences	22	RF, point-by-point
Deneke et al. (18)	88	DWI sequences	51	RF, point-by-point (46.6%)/Cryobaloon, single shot (22.7%)/Laserbaloon, single shot (30.7%)
Di Biase et al. (19)	428	DWI EPI sequences	42	RF, point-by-point
Deneke et al. (20)	43	DWI sequences	26	RF, point-by-point
Wissner et al. (21)	86	DWI sequences	21	RF, point-by-point (25.6%)/Cryobaloon, single shot (23.2%)/Laserbaloon, single shot (51.2%)
Von Bary et al. (22)	52	DWI/FLAIR sequences	54	RF, point-by-point (90.4%)/Cryobaloon, single shot (9.6%)
Bergui et al. (23)	927	DWI sequences	164	RF, point-by-point/Cryobaloon, single shot^a^
Nakamura et al. (25)	160	DWI/FLAIR sequences	64	RF, point-by-point/Cryobaloon, single shot^a^
Nagy-Balò et al. (26)	27	DWI/FLAIR sequences	11	RF, point-by-point
Miyazaki et al. (27)	256	DWI/FLAIR sequences	180	RF, point-by-point/Cryobaloon, single shot^a^
Keçe et al. (28)	70	DWI/FLAIR/TSE sequences	18	RF, point-by-point
Yu et al. (29)	55	DWI sequences	106	RF, point-by-point
Malikova et al. (30)	30	DWI/FLAIR sequences	3	RF, point-by-point

FLAIR, fluid attenuated inversion recovery; EPI, echo-planar imaging; RF, radiofrequency.

^a^ Percentages not available.

**Figure 2:**
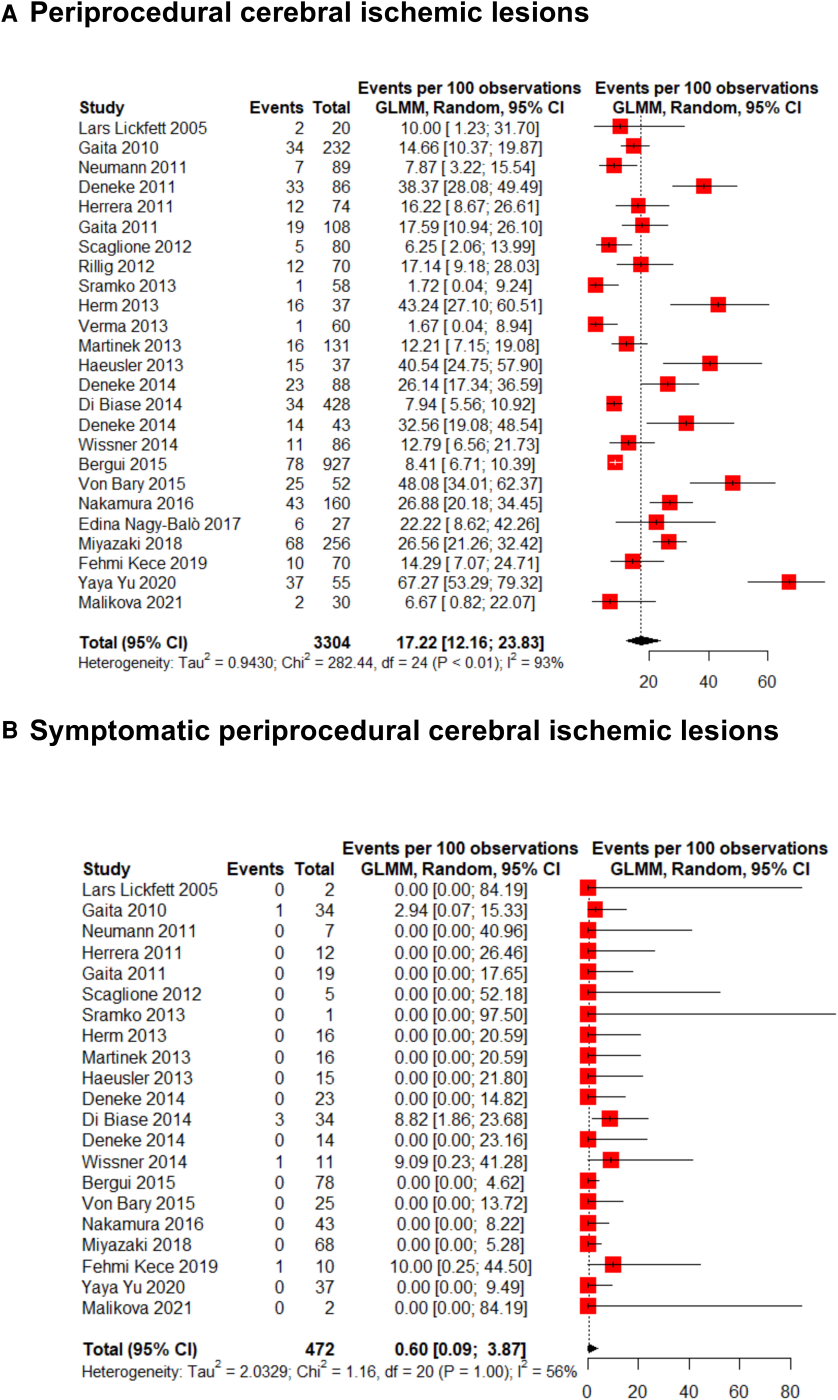
Forest plot for (**A**) overall and (**B**) symptomatic periprocedural cerebral ischemic lesions incidence.

The incidence of new ischemic cerebral lesions in patients that had undergone AF transcatheter ablation was 17.2% (95% CI 12.2%–23.8%; *I*^2^: 93%) (Figure 2A). Among these lesions, only a minimal proportion had ischemic symptoms [0.60% (95% CI 0.09%–3.9%; *I*^2^: 56%)] (Figure 2B).

**Figure 3:**
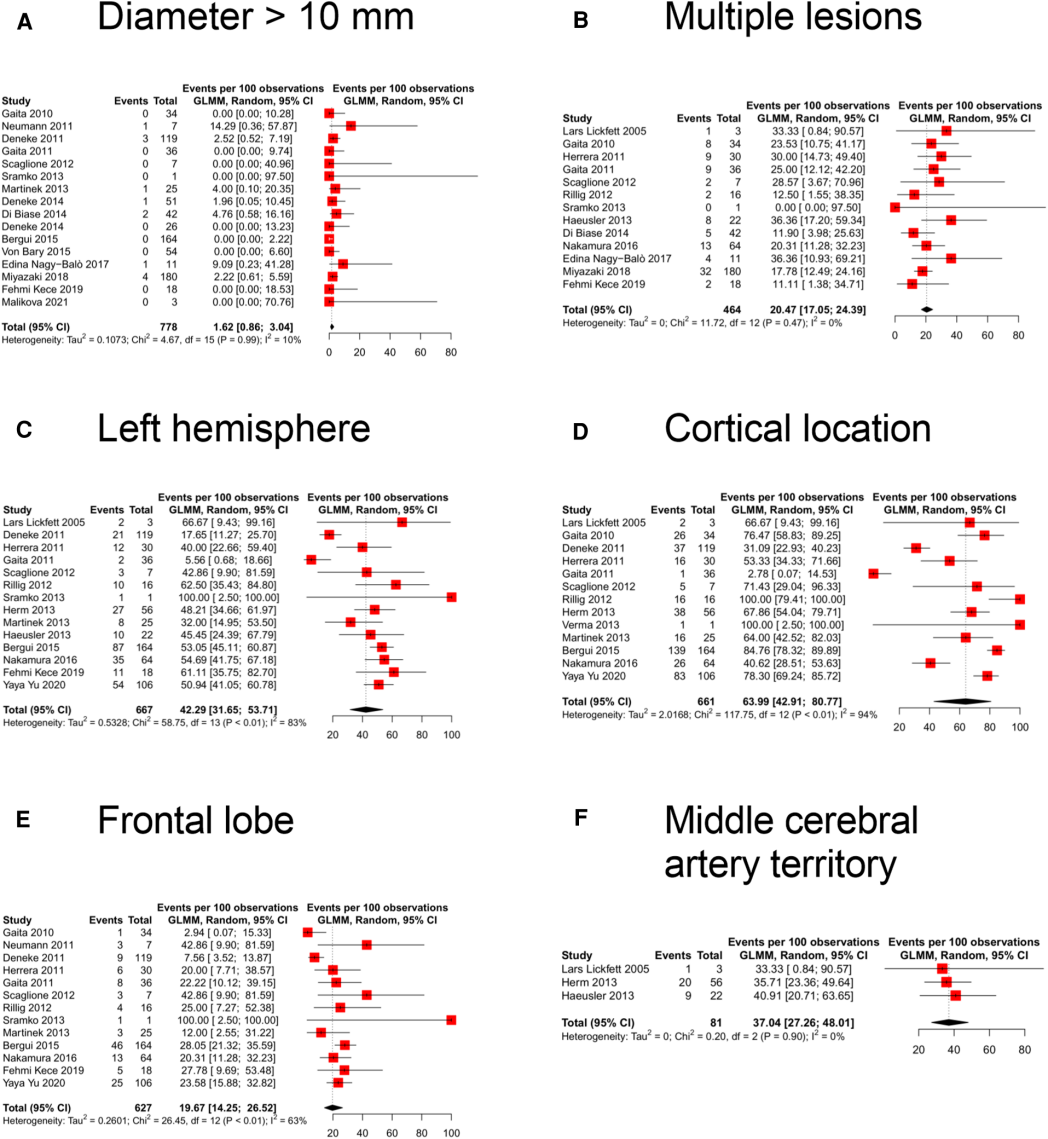
Forest plot of the different neuroimaging features: (**A**) diameter more than 10 mm, (**B**) multiple lesions, (**C**) left hemisphere location, (**D**) cortical location, (**E**) frontal lobe location, and (**F**) middle cerebral artery territory location.

Concerning the neuroimaging features of the new cerebral ischemic lesions, the size, reported in 16 studies (evaluating 778 lesions), had a diameter of above 10 mm in 1.6% (95% CI 0.9%–3.0%; *I*^2^: 10%) of the cases (Figure 3A). Multiple lesions, described in 13 studies (464 lesions), were reported in 20.5% (95% CI 17.1%–24.4%; *I*^2^: 0%) of the scans (Figure 3B). The pooled prevalence of left hemisphere location, reported in 14 studies (667 lesions), was 42.3% (95% CI 31.7%–53.7%; *I*^2^: 83%) (Figure 3C). The cortical location (reported in 13 studies, 661 lesions) showed the highest pooled prevalence [64.0% (95% CI 42.9%–80.8%; *I*^2^: 94%); Figure 3D] while lesions were, instead, subcortical (5 studies, 266 lesions) or cerebellar (12 studies, 627 lesions) in 25.5% (95% CI 7.1%–60.7%; *I*^2^: 91%; Supplementary Figure S1) and 15.2% (95% CI 9.7%–23.1%; *I*^2^: 75%; Supplementary Figure S2) of the cases, respectively. The pooled prevalence of frontal lobe location (13 studies, 627 lesions) was 19.7% (95% CI 14.3%–26.5%; *I*^2^: 63%; Figure 3E), while parietal (12 studies, 595 lesions), occipital (12 studies, 593 lesions), and temporal (10 studies, 537 lesions) lobes were involved in 17.1% (95% CI 9.3%–29.5%; *I*^2^: 87%; Supplementary Figure S3), 12.1% (95% CI 8.9%–16.1%; *I*^2^: 29%; Supplementary Figure S4), and 8.6% (95% CI 3.5%–19.8%; *I*^2^: 79%; Supplementary Figure S5) of the scans, respectively. The vascular territory of the MCA was the most commonly involved (3 studies, 81 lesions), reported in 37.0% (95% CI 27.3%–48.0%; *I*^2^: 0%; Figure 3F) of the cases. ACA (3 studies, 242 lesions), PCA (3 studies, 242 lesions), and border zone (3 studies, 108 lesions) territories were involved in 28.5% (95% CI 23.2%–34.5%; *I*^2^: 0%; Supplementary Figure S6), 28.1% (95% CI 22.8%–34.1%; *I*^2^: 0%; Supplementary Figure S7), and 22.9% (95% CI 8.5%–48.8%; *I*^2^: 81%; Supplementary Figure S8) of the scans, respectively.

## Discussion

**Figure 4:**
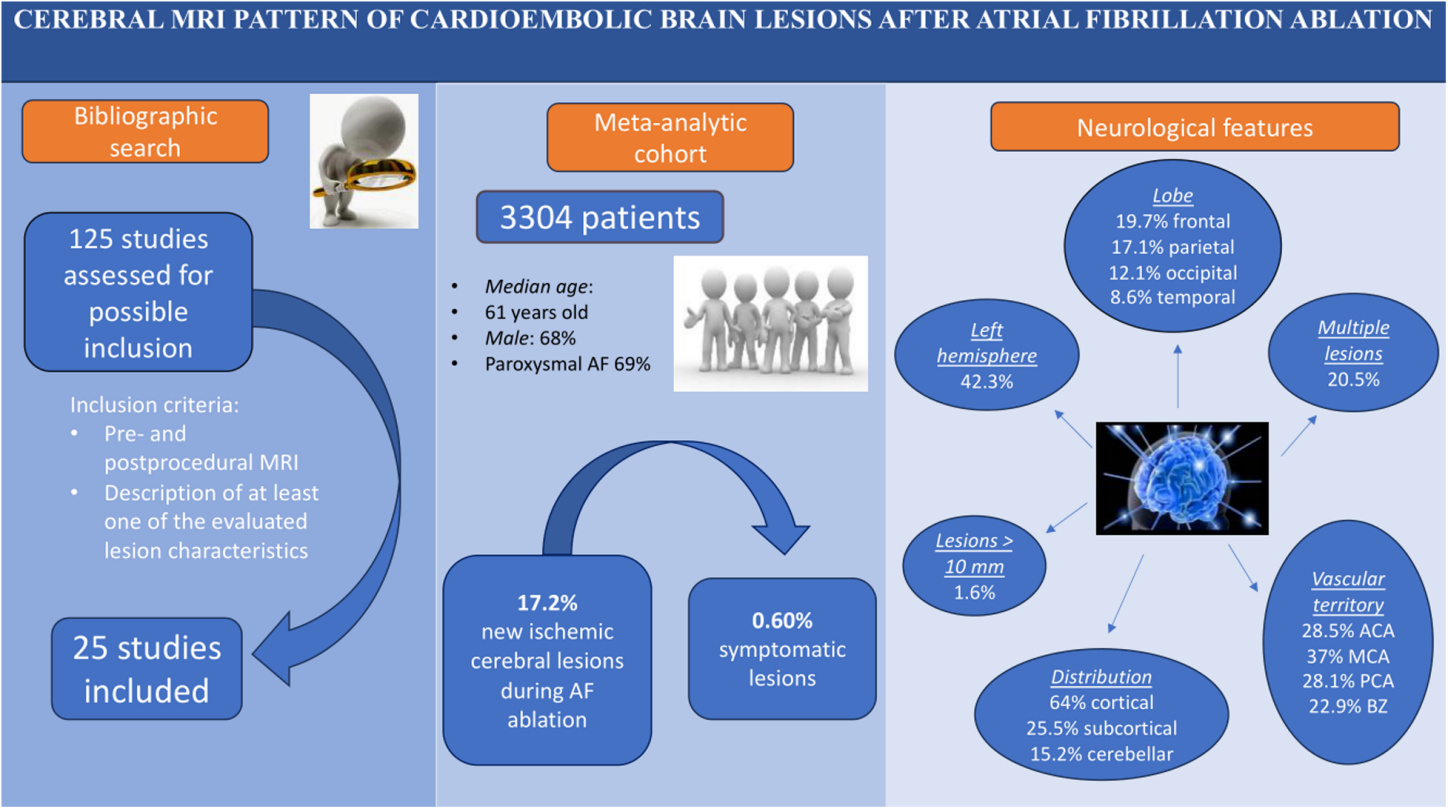
Graphical abstract.

The main findings of the present analysis are as follows (Figure 4—Graphical Abstract):

• Among the proportion of patients presenting a cerebral ischemic lesion at cerebral MRI after AF transcatheter ablation (17%), 0.6% are symptomatic from a neurological standpoint.
• These cardioembolic cerebral lesions are generally balanced between the right and the left sides and are ubiquitously detected in all cerebral lobes; the lesions are typically small (diameter less than 10 mm), generally single (multiple only in about 20% of the cases), and they preferentially affect the cerebral cortex (in nearly two-third of the cases) of the MCA vascular territory.

Determining the underlying cause of an acute stroke is important not only to guide patient's immediate management but also to prevent new events, given that stroke recurrence is strongly related to its specific etiology (38–40). Imaging has a primary role in early diagnosis of strokes: in fact, some patterns of brain infarction may suggest a specific cause. In particular, regarding ESUS, early identification of a potential cause underlying the ischemic event might be of paramount importance, since it could expedite the clinical decision process leading to the adoption of therapeutic strategies, such as initiation of oral anticoagulation in case of an AF-related genesis. Moreover, considering that patients with AF frequently present asymptomatic cerebral lesions (41), keeping in mind that AF-related subclinical lesions might be due to several mechanisms (42–47), recognizing a certified neuroimaging pattern suggestive of subclinical AF-related lesions might even help prevent the occurrence of clinically relevant events.

Despite these potential benefits, a systematic description of the neuroimaging features associated with a cardioembolic genesis of ischemic strokes is presently lacking. For this purpose, AF transcatheter ablation can be regarded as an *in vivo* model of cardioembolic lesions, used to derive a neuroimaging “fingerprint” of typical cardioembolic lesions. In fact, during an uncomplicated transcatheter AF ablation the patient does not experience hemodynamic compromise, making a hypoperfusive genesis (due to transient reduction of cardiac output) of new cerebral ischemic lesions not plausible. Other cardioembolic models, such as cardiac surgery [during which hypotensive episodes might occur (48)] or transcatheter aortic valve replacement [the rapid ventricular pacing performed during valve deployment temporarily reduces cardiac output (49)], certainly do not share this feature.

The present analysis suggests that typical cardioembolic lesions, such as occurring during an AF ablation procedure, tend to be located at a cortical level and, particularly, in the vascular territory of the middle cerebral artery. Cerebral lesions related to a hypoperfusive genesis, instead, more commonly are subcortical and determine watershed infarcts (50–52). Watershed infarcts occur at the border between cerebral vascular territories where the tissue is furthest from arterial supply and thus most vulnerable to hypotension and hypoperfusion, or might exacerbate embolism-related damage (e.g., delayed embolism “washout,” impaired perfusion of ischemic penumbra) (53). In any case, this neuroimaging “fingerprint” differs quite clearly from that of cerebral lesions related to a cardioembolic genesis.

### Limitations

The analysis is limited by the inherent limitations of a meta-analysis. In particular, although all studies performed diffusion-weighted imaging sequences to detect new cerebral lesions, hidden technicalities across the different studies cannot be excluded. Moreover, the present results might apply prevalently to a population of paroxysmal AF patients undergoing catheter ablation. In addition, although we selected a cut-off value of 10 mm to distinguish between smaller and larger lesions, we cannot exclude that different cut-offs (e.g., 3 or 5 mm) might be more appropriate and provide different trends. Finally, we cannot directly exclude that the populations included in studies from the same Research Groups might present partial overlaps.

## Conclusions

In conclusion, by thoroughly assessing incidence and neuroimaging features of cerebral ischemic lesions following AF transcatheter ablation, it emerges that in predominance they consist of small (<10 mm) cortical lesions, almost ubiquitous in all cerebral lobes and in both hemispheres, prevalent in the territory of the middle cerebral artery.

The present analysis supports the existence of an, at least, suspicious neuroimaging “fingerprint” of cardioembolic brain lesions. If confirmed in specifically designed studies, specific neuroimaging features in *de novo* cerebral lesions would rapidly prompt a tailored clinical management, shortening time to diagnosis of the underlying etiology, and, potentially, preventing recurrences.

## Data Availability

The raw data supporting the conclusions of this article will be made available by the authors, without undue reservation.
