# Supplementary material for: MRI pattern characterization of cerebral cardioembolic lesions following atrial fibrillation ablation
Source: Front Cardiovasc Med. 2024 Jan 24;11:1327567. doi: 10.3389/fcvm.2024.1327567 (PMC10847299; doi:10.3389/fcvm.2024.1327567)

**MRI PATTERN CHARACTERIZATION OF CEREBRAL CARDIOEMBOLIC LESIONS FOLLOWING ATRIAL FIBRILLATION ABLATION**

*Supplementary Materials*

**Supplementary Figure 1.** Forest plot for subcortical lesion location.


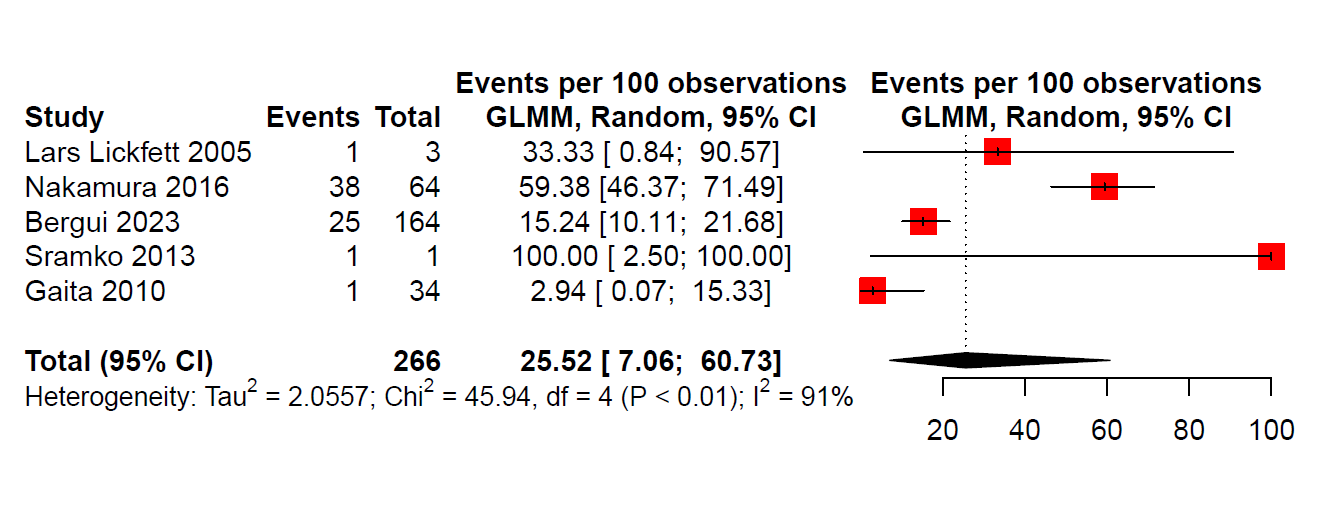


**Supplementary Figure 2.** Forest plot for cerebellar lesion location.


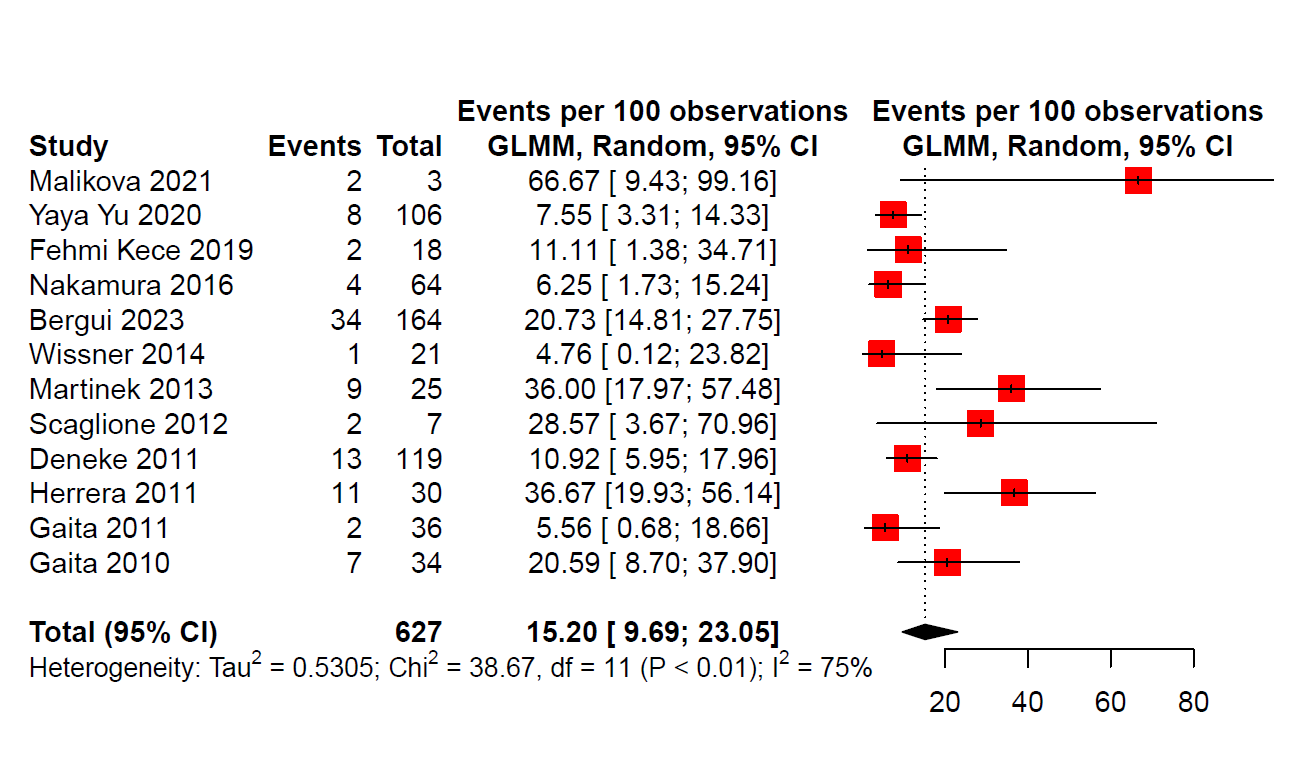


**Supplementary Figure 3.** Forest plot for parietal lobe location.


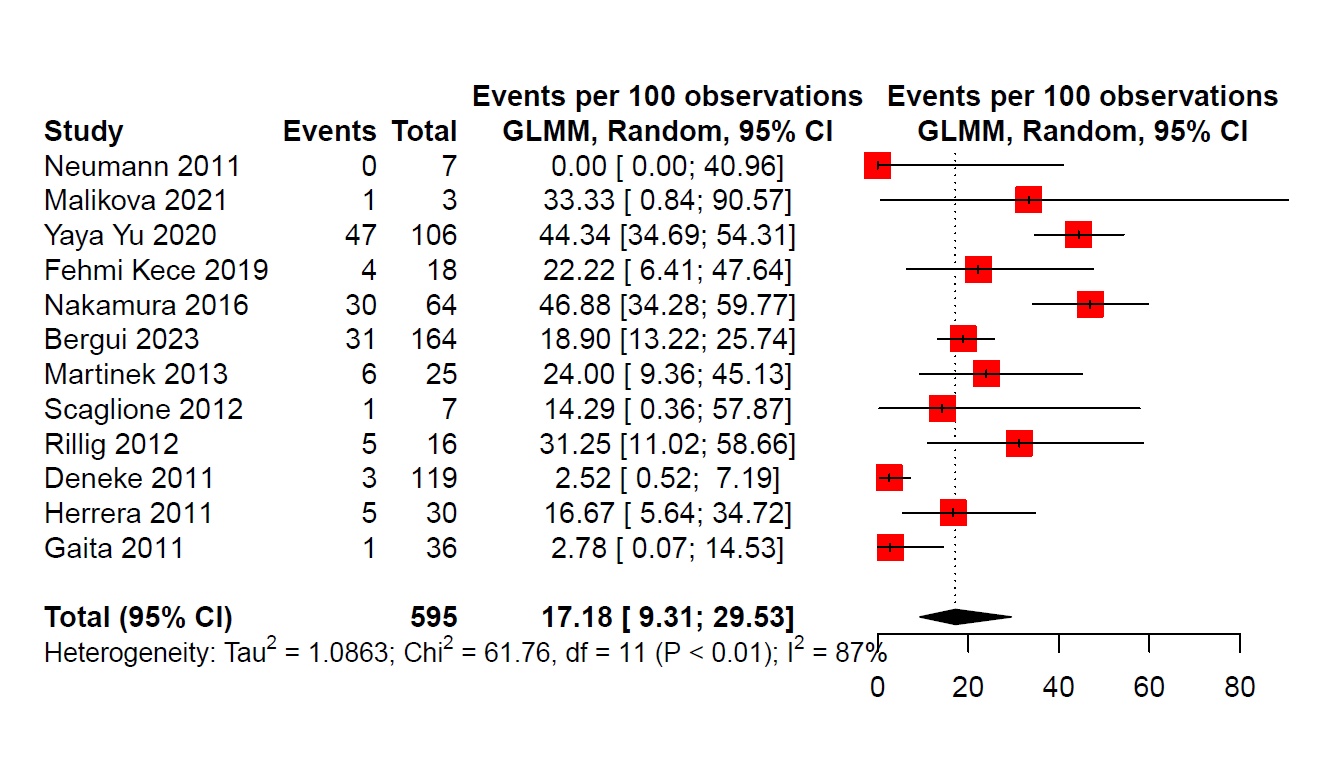


**Supplementary Figure 4.** Forest plot for occipital lobe location.


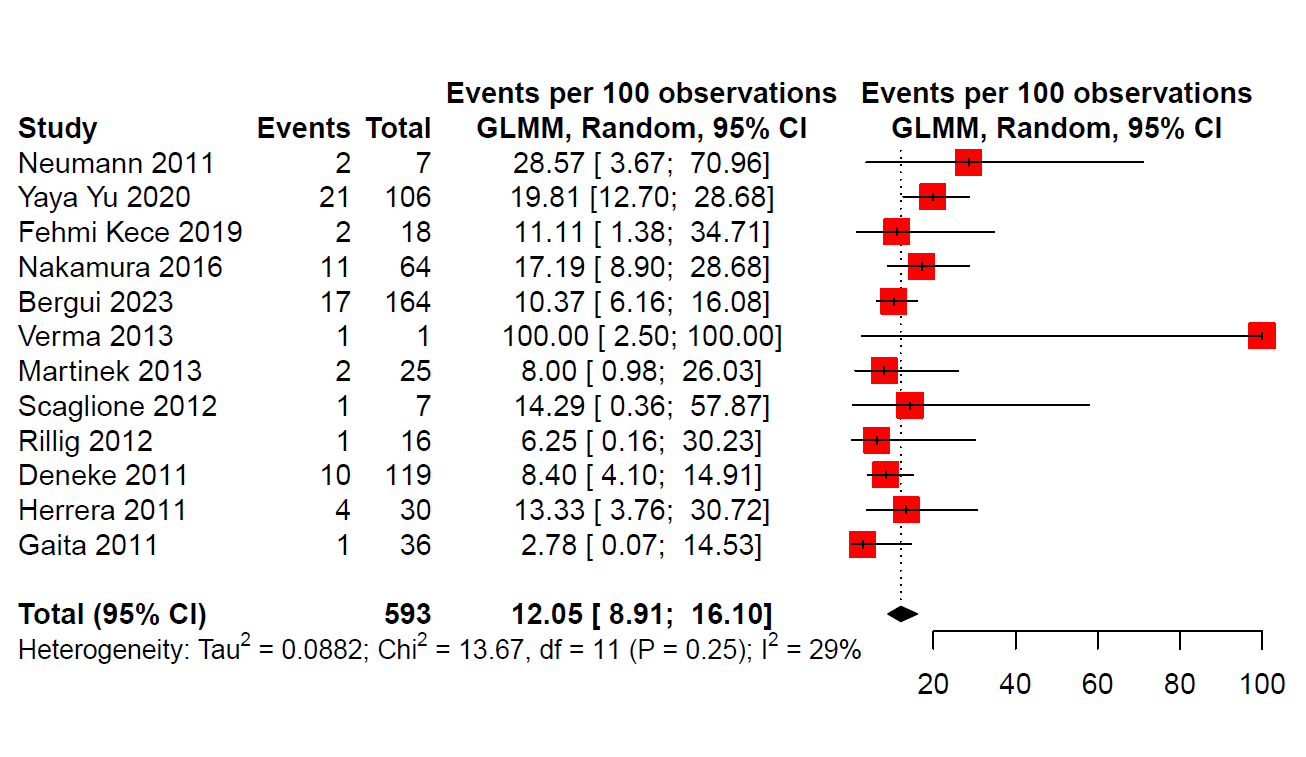


**Supplementary Figure 5.** Forest plot for temporal lobe location.


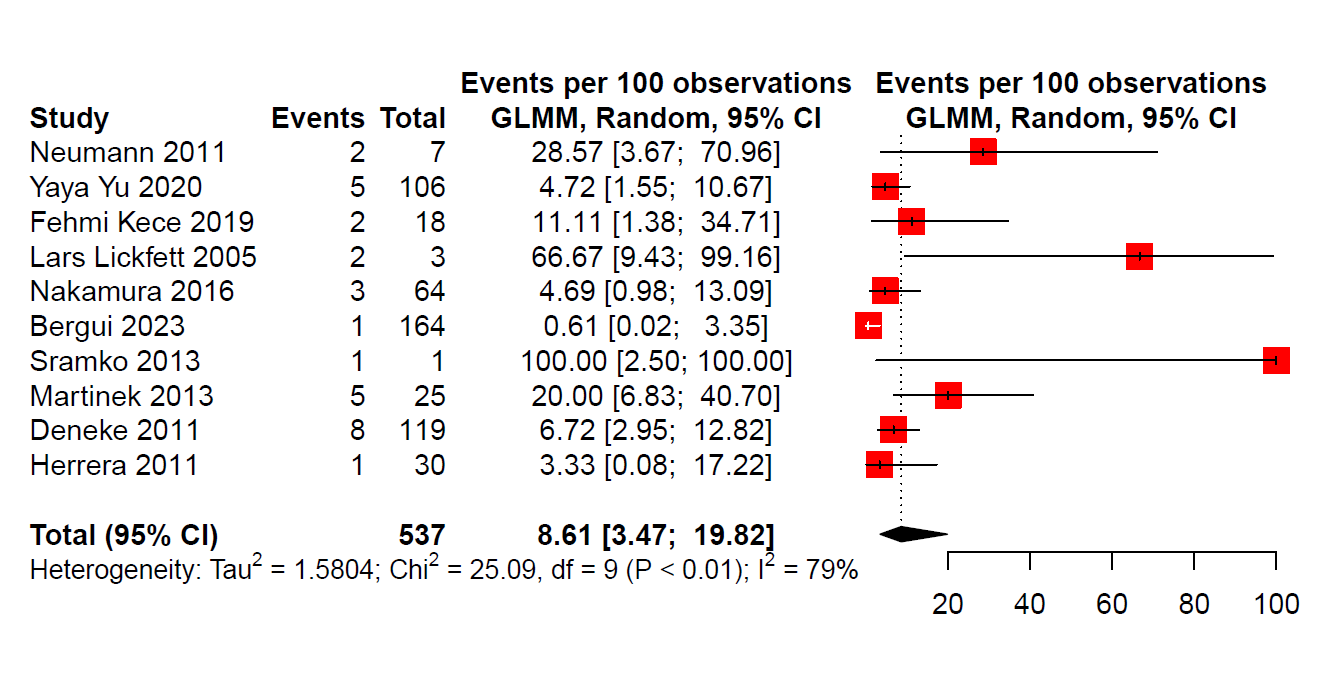


**Supplementary Figure 6.** Forest plot for anterior cerebral artery territory location.


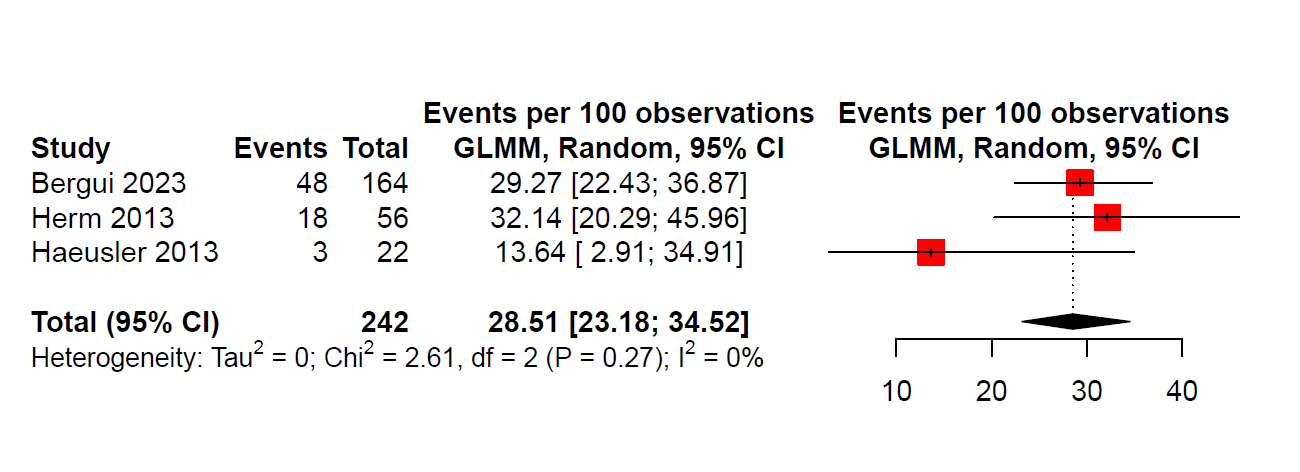


**Supplementary Figure 7.** Forest plot for posterior cerebral artery territory location.


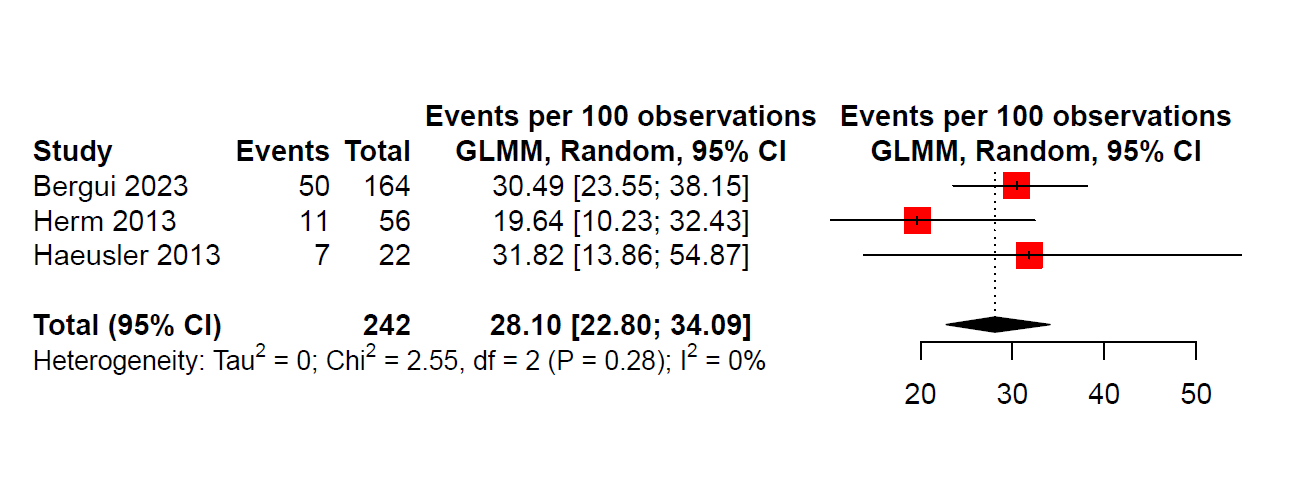


**Supplementary Figure 8.** Forest plot border zone territory location.


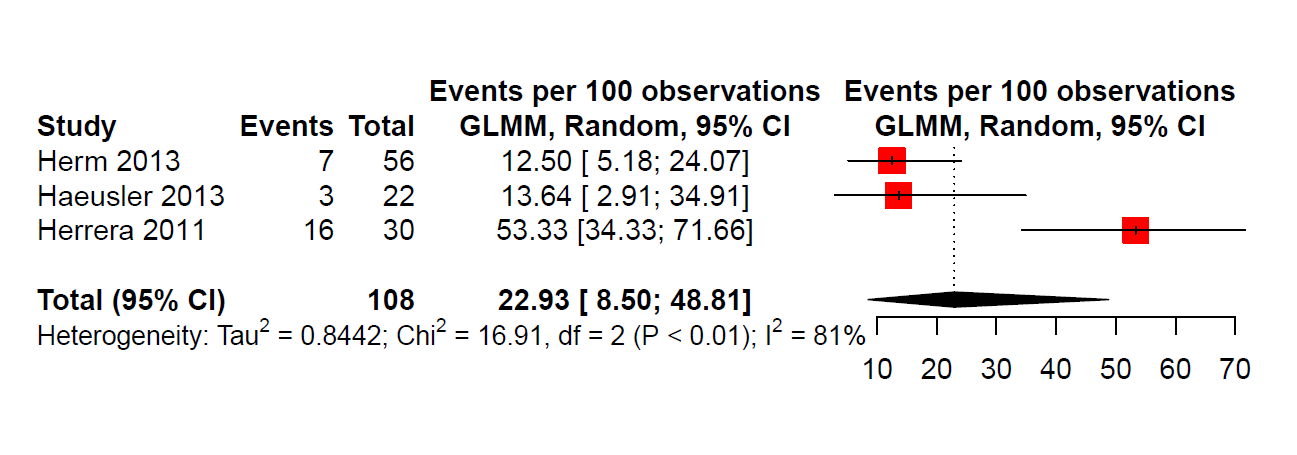

Supplement: Supplementary file 1 [file Datasheet1.docx]
